# Integrating large language models into medical undergraduate laboratory course to enhance bioethical competence: a quasi-experimental study

**DOI:** 10.3389/fmed.2025.1745975

**Published:** 2026-02-05

**Authors:** Yue Wang

**Affiliations:** Department of Cell Biology and Genetics, The School of Basic Medical Sciences, Fujian Medical University, Fuzhou, Fujian, China

**Keywords:** artificial intelligence, bioethics, large language model, medical cell biology laboratory course, undergraduate medical education

## Abstract

**Objective:**

This study investigates the integration of different large language models (LLMs) into the Medical Cell Biology Laboratory Course (MCBLC) to enhance bioethics training for undergraduate medical students in China. It further compares the effectiveness of these LLMs in improving teaching outcomes and student learning performances. Key challenges encountered during implementation were identified, and potential strategies to address them were also explored.

**Methods:**

First-year undergraduate medical students from three medical majors were assigned to five groups. The study involved three phases: instructor-led course introduction, LLM-assisted experimental practice addressing procedural, conceptual, and psychological challenges, and post-training evaluation via questionnaires and blind-graded laboratory reports. Four domestic robust LLMs (DeepSeek, Doubao, KIMI, ChatGLM) were compared to assess their impact on bioethics integration, instructional effectiveness, and student learning outcomes, while documenting students' perceptions and concerns regarding LLM use.

**Results:**

The study demonstrated that all four LLMs supported first-year undergraduate medical students in consolidating foundational knowledge, enhancing bioethics proficiency during laboratory practice, and developing critical competencies for future physicians. Questionnaires from 86 students across three majors indicated generally high satisfaction. For Medical Imaging Technology students, DeepSeek (mean 4.3, SD 0.7) and KIMI (mean 4.3, SD 0.8) were rated significantly higher than Doubao (mean 3.9, SD 0.7) and ChatGLM (mean 3.3, SD 0.6). KIMI was also preferred among Health Surveillance and Quarantine (mean 4.4, SD 0.5) and Medical Prevention (mean 4.5, SD 0.5) students. Nevertheless, students expressed concerns regarding potential academic inaccuracies, bias, and possible impact on independent thinking.

**Conclusions:**

This study suggested that recent LLMs, particularly KIMI and DeepSeek, may support integrating bioethics into undergraduate medical laboratory courses in a university in China. By assisting students in accessing information, reflecting on ethical issues, and navigating practical challenges, these tools can facilitate learning and foster ethical awareness, competent future physicians. These findings, as an initial exploration and context-specific, indicate that LLMs may support bioethics learning in undergraduate medical laboratory courses and help foster ethically aware, competent future physicians.

## Introduction

Bioethics-oriented basic medical education has emerged as an influential paradigm in undergraduate medical training, exerting a significant impact on students during the early stages of their medical studies (1, 2). By integrating ethical, humanistic, existential and inclusive perspectives into the foundational medical curricula, it plays a crucial role in shaping students' professional identity and moral outlook (3). By fostering bioethical sensitivity among medical undergraduates in both clinical practice and bioethical research (4), it lays a foundation for further engagement. Students are then immersed in authentic bioethical cases, gaining the ability to manage psychological stress and navigate ethical dilemmas in academic and clinical environments (5). This ultimately equips future medical professionals to shoulder the responsibility of defining and upholding bioethical principles for the benefit of humanity and the global community (6, 7).

As biomedical science advances rapidly, the ethical challenges accompanying technological innovation have grown increasingly complex. A key issue in medical education is how to integrate bioethical principles effectively into foundational medical sciences (8). Genetic testing and result disclosure exemplify this need: while screening can prevent the transmission of sex-linked diseases, misuse for non-medical purposes such as sex selection may distort population balance and undermine societal ethics (9, 10). In biomedical research, the boundaries of genetic intervention remain debated. Gene therapy has achieved significant progress, as evidenced by FDA-approved treatments using lentiviral vectors and CRISPR/Cas9 for transfusion-dependent β-thalassemia (11, 12). Nevertheless, unethical practices such as human germline editing demonstrate how scientific advances can outpace moral oversight (13). Beyond the laboratory, healthcare professionals encounter complex ethical dilemmas in both study and practice, including asymmetries in informed consent, moral stress in emergencies, and conflicts in treatment decision-making (14). These challenges underscore an urgent educational responsibility, that is, medical undergraduates must be prepared early with solid bioethical knowledge, reflective skills, and psychological resilience. Such preparation enables them to navigate ethical complexities competently and to participate responsibly in the development and implementation of evolving standards that protect human dignity and promote societal welfare.

The growing complexity of ethical dilemmas in modern medicine has prompted educators to seek innovative strategies for cultivating bioethical awareness among undergraduates. Among existing strategies, institutional workshops or specialized training provide in-depth instruction on bioethical issues. However, they depend heavily on organizational support and lack flexibility (15). Similarly, integrating ethics cases or current affairs into courses leverages individual teaching expertise but often offers a single-dimensional perspective, insufficient for students' multifaced ethical reflection (16). In the context of LLM-driven innovation, advanced computational power and vast knowledge repositories allow LLMs to provide efficient, holistic, and readily accessible solutions (17). These capabilities have been widely applied across education, medicine, engineering, and the social sciences, tackling complex challenges with remarkable breadth and precision (18–21). In this study, LLMs were explored as a dynamic platform within a medical cell biology course, employing a student-teacher collaborative approach. By promoting active engagement with bioethical principles, this method aims to develop undergraduates who combine competence with ethical awareness, while providing a scalable and adaptable model to enhance medical ethics education and support responsible, ethically informed practice.

## Methods

### Study overview

This study adopted a quasi-experimental design with student self-selection to explore whether large language models (LLMs) could promote medical undergraduates' understanding of bioethics-oriented values in a cell biology laboratory course. The instructional intervention focused on a laboratory task involving the examination of sperms to observe chromosomes in germ cells, a topic selected for its biological relevance as well as its ethical implications in medical education.

The study was conducted in three sequential phases. In the first phase, participating students received a standardized introduction covering the course structure, learning objectives, background concepts, experimental procedures, and report writing requirements from the instructor. Students were informed of multiple learning options, including the use of one of four LLM tools (DeepSeek, Doubao, KIMI, or ChatGLM) or the option of not using any LLM during the laboratory course.

During in-class laboratory activities, students who chose to use LLM tools were permitted and encouraged to access them using personal mobile phones, tablets or laptop computers to support their learning and ethical reflection. Based on their personal preferences, students voluntarily selected their preferred learning approach and were subsequently categorized into five groups: four LLM-assisted learning groups (one for each LLM tool) and one non-LLM group. To avoid extremely large or small group sizes that could affect instructional feasibility and data analysis, the instructor provided limited informational guidance regarding approximate group sizes during the selection process. Final group decisions remained entirely voluntary, and no student was reassigned by the instructor. No random allocation or compulsory reassignment was applied.

**Figure 1:**
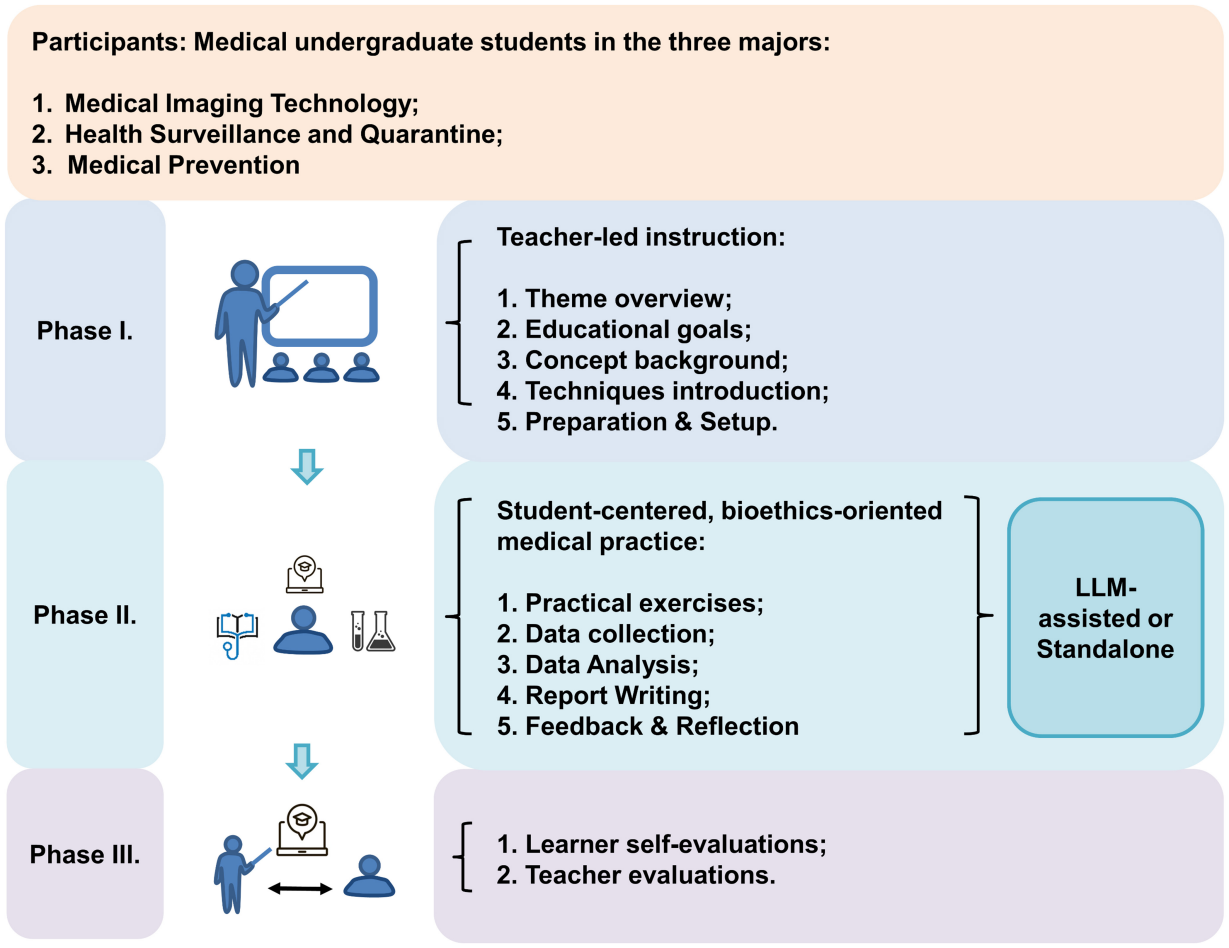
A schematic overview of the study on LLM-assisted integration of bioethical principles into the undergraduate cell biology laboratory course and its impact on educational outcomes.

In the final phase, all students completed a laboratory report and a post-course questionnaire reflecting on their learning experience, ethical engagement, and perceptions of LLM-assisted learning. Laboratory reports were collected and graded by the instructors, who also provided qualitative evaluations. Students' learning outcomes and feedback were then compared across the five groups to examine whether and how the use of different LLM tools influenced ethical awareness, concerns related to LLM platforms, and perceived learning effectiveness in the context of integrating bioethical principles into basic medical education (Figure 1).

### Participants

A total of 86 medical undergraduates participated in this study. All participants were enrolled in the cell biology laboratory course during the 2025 academic year and were drawn from three majors: Medical Imaging Technology (*n* = 31), Preventive Medicine (*n* = 28), and Health Surveillance and Quarantine (*n* = 27). These majors were selected because each represents a distinct interdisciplinary orientation within medical education. General demographic information of the participants is summarized in Tables 1, 2.

**Table 1:** General information of all participants from the three majors.

**General information of participants**				
**Items**/**Major**		**Medical imaging technology**	**Medical prevention**	**Health surveillance and quarantine**
Subcategories	Total number of participants enrolled	31	28	27
Gender	Male	15 (48.39%)	10 (35.71%)	11 (40.74%)
	Female	16 (51.61%)	18 (64.29%)	16 (59.26%)
Age	< 15	0 (0%)	0 (0%)	0 (0%)
	15–25	31 (100%)	28 (100%)	27 (100%)
	>25	0 (0%)	0 (0%)	0 (0%)
Degree	First-year undergraduate students (regular track)	30 (96.77%)	28 (100%)	27 (100%)
	First-year undergraduate students (repeat track)	1 (3.23%)	0 (0%)	0 (0%)

^*^Values are shown as number of students, with percentages in parentheses representing the proportion of participants within each major.

**Table 2:** Overview of academic background of the students across three majors.

**Overview of academic background across three majors**				
**Items**/**Major**		**Medical imaging technology**	**Medical prevention**	**Health surveillance and quarantine**
Previous and current coursework	Advanced mathematics for medicine	1	1	1
	Medical physics	1	1	1
	College english	1	1	1
	History	1	1	1
	Physical education	1	1	1
	Mental health education	1	1	1
	Ideological and moral education	1	1	1
	Organic chemistry	1	1	1
	Systematic anatomy	1	1	1
	Cell biology	1	1	1
	Physiology	1	0	0
	Biochemistry and molecular biology	0	1	1
	Fundamentals of computer applications	0	1	1
	Histology and embryology	1	1	0

^*^Participants' course exposure in the three majors was coded as a binary variable (1 = completed or currently enrolled, 0 = not yet taken but planned to be taken in the future).

Medical Imaging Technology integrates foundational medical knowledge with engineering principles, emphasizing cross-disciplinary competencies required in modern diagnostic practice (22). Preventive Medicine combines medical science with social science perspectives, reflecting the integration of theoretical knowledge and applied approaches in public health and disease prevention (23). Health Surveillance and Quarantine draws on medicine and natural sciences, requiring a comprehensive understanding of both theoretical frameworks and practical applications within broader biological and medical contexts (24).

### Selected LLMs and modes of interaction

Large language models (LLMs) are defined as advanced text-based machine learning models. They have been trained on extensive multilingual corpora to generate and analyze natural language. In the context of this educational reform study, LLMs were used as interactive tools that assist undergraduate students in retrieving, understanding, and reflecting on bioethical knowledge within the cell biology laboratory course. Four prominent LLMs in China were evaluated in this study. DeepSeek, developed as a free, open-source platform, excels in generating clinical materials and has demonstrated strong performance in national medical examinations (25). Doubao, created by ByteDance, is trained on extensive Chinese social media datasets and performs well in addressing routine queries (26). KIMI, developed by Moonshot AI, has been applied in clinical contexts for generating case descriptions due to its scientific rigor and reliability (27). ChatGLM, developed by Tsinghua University and collaborators, is suitable for both daily use and academic research (28). We assessed how these LLMs can facilitate the integration of bioethical education into medical laboratory courses using student surveys and teacher evaluations of laboratory reports.

The versions of the four LLMs used in this study were DeepSeek-R1, Doubao-1.5 Pro, KIMI K2 series, and ChatGLM/GLM series. During the laboratory course, students accessed these platforms on personal devices, such as cell phones, tablets, or laptops via web-based interfaces. Interactions were conducted in Chinese, with typical factual or explanatory prompts, such as “What is the process of sample collection?”, simulating realistic laboratory queries. All LLMs adhered to the same local internet security and data protection regulations. System messages or guardrails were not standardized beyond the publicly available interfaces, reflecting natural usage in educational settings.

### Evaluation metrics

Both the student questionnaire and instructor evaluations used a standardized five-point Likert scale for assessment. Each item required respondents to select one of five options, with no alternative or intermediate values allowed. The scale was defined as follows: 1 = poor or very dissatisfied, 2 = limited or slightly dissatisfied, 3 = fair or basically satisfied, 4 = good or satisfied, and 5 = excellent or very satisfied. Respondents were required to choose a single score for each item, ensuring that all ratings were constrained to this 1–5 scale. This approach allowed for consistent quantification of student performance, engagement, and instructor evaluations while maintaining clarity and standardization in the assessment process.

### Instructor rating of laboratory reports

Laboratory reports were completed following a standardized format that included five predefined sections: experimental objectives, materials and reagents, procedures, observations, and key precautions. Lab reports were evaluated using a 5-point scale (1 = poor to 5 = excellent), and the scores were combined to produce an overall rating reflecting the quality and completeness of the report. All reports were assessed by a single course instructor who was blinded to students' group assignments. The evaluation followed a structured approach based on the predefined report components and aligned with the instructional objectives of the cell biology laboratory course. Although inter-rater reliability could not be assessed due to the use of a single rater, this approach was intended to maintain consistency in scoring across all student groups.

### Statistical analysis

All statistical analyses were conducted using GraphPad Prism 7.0 and SPSS 26.0. Continuous variables were summarized using means and standard deviations, and categorical variables were summarized using frequencies and percentages. Internal consistency of the measurement instruments was assessed using Cronbach's alpha. Differences among the student groups were analyzed using one-way analysis of variance (ANOVA). The assumptions of normality and homogeneity of variances were checked before analysis. When ANOVA indicated a significant group effect, *post-hoc* pairwise comparisons were performed using Tukey's test. For all comparisons, *F* values, *p*-values, adjusted *p*-values, and 95% confidence intervals (CI) were reported. The significance level was set at α = 0.05. Effect sizes were calculated to complement *p*-values, providing an estimate of the magnitude of observed differences. Graphs and figures were generated using GraphPad Prism 7.0.

## Results

### Evaluation of the effectiveness of LLMs in integrating bioethical principles into medical education, using both student self-assessments and instructor grading

**Figure 2:**
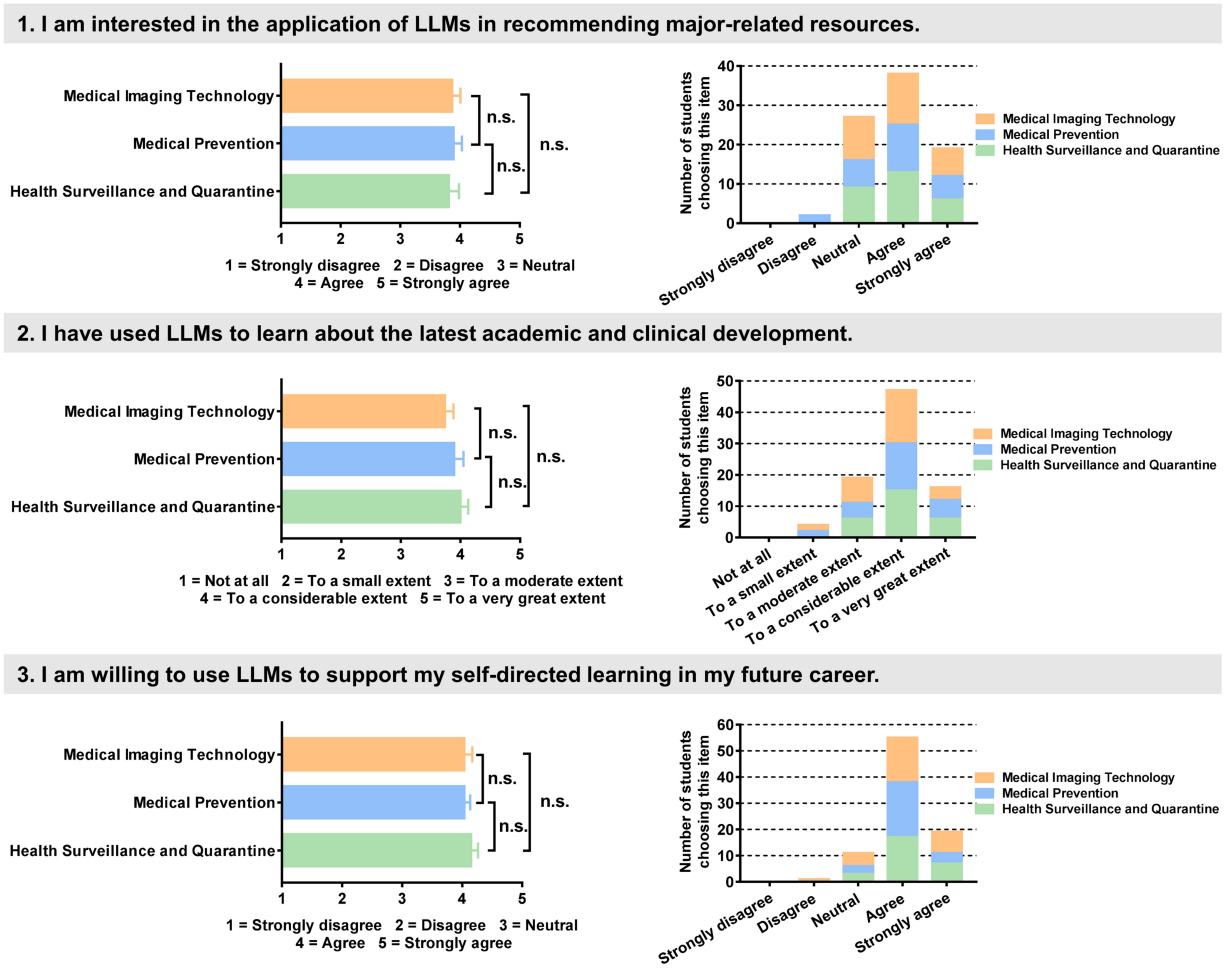
Evaluation of students' interest in, experience with and willingness to use LLMs (one-way ANOVA with *post-hoc* Tukey's test, *p < 0.05, n.s. = no significant difference).

The study included medical undergraduate students enrolled in courses during the 2025 academic year from three majors: Medical Imaging Technology, Medical Prevention, and Health Surveillance and Quarantine. Students' interest in, experience with, and willingness to use LLMs were assessed via the course questionnaire using a 1–5 Likert scale, and no significant differences were observed among students from the three majors (Figure 2).

**Figure 3:**
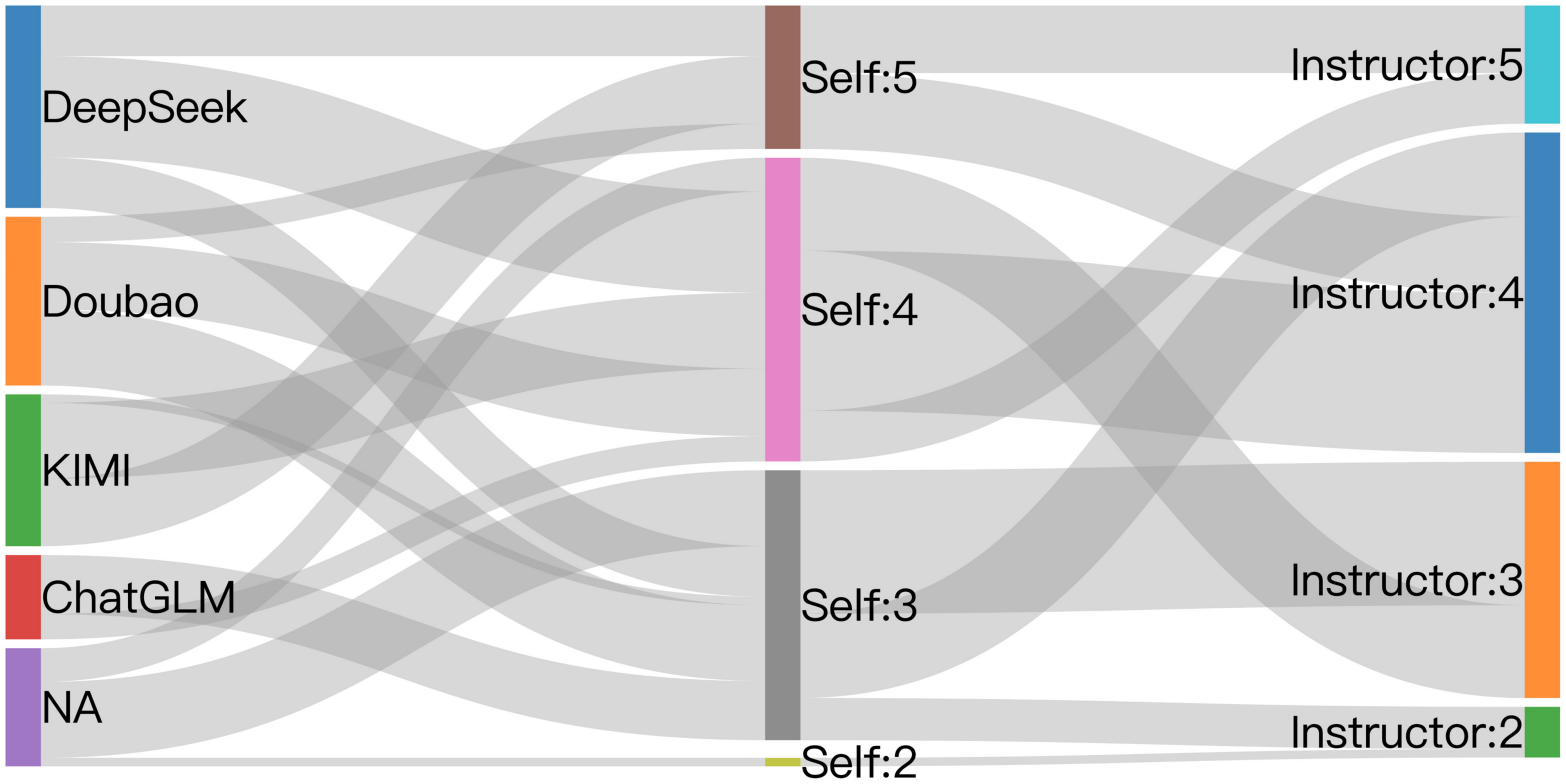
Overall assessment of LLM use in integrating bioethical principles into undergraduate medical education. Sankey diagram with three vertical columns: selected platforms **(Left)**, students' self-evaluation of platform effectiveness **(Middle)**, and teachers' assessment scores of the students' lab reports **(Right)**.

In the laboratory course, students are expected to learn experimental design, principles, techniques, operations, and data analysis, and to summarize their findings in a laboratory report. These reports provide an objective measure of learning outcomes and help students consolidate practical knowledge, reflect actively, and organize theoretical and operational skills systematically. Preparing the report completes a full learning cycle and lays a foundation for future scientific inquiry. In this study, course effectiveness was evaluated from both instructor and student perspectives through instructor grading based on predefined criteria and student self-assessments, including experiences with or without LLM-assisted support. We collected overall assessments of using LLMs in bioethics-oriented education in medical cell biology laboratory course from 86 students across the three majors, using a five-point Likert scale ranging from 1 (not satisfied) to 5 (very satisfied) (Figure 3, the central lane). The self-evaluation data were integrated with instructors' grading of students' laboratory reports (assessed on a five-point scale ranging from 1 = poor to 5 = excellent). To clearly demonstrate the association among platform selection, students' perceived effectiveness, and their academic performance, a comprehensive Sankey diagram was generated, linking students' evaluations with instructors' assessments. The results clearly show that, among the four LLMs, both DeepSeek and KIMI received the highest levels of overall satisfaction from students. Meanwhile, Doubao and ChatGLM received moderate evaluations. Students reporting higher satisfaction with LLMs generally achieved higher grades, suggesting alignment between self-reported evaluations and objective performance. Conversely, students who did not use LLM tools gave lower evaluations and received lower lab grades, indicating that LLM use may positively influence both learning outcomes and student satisfaction (Figure 3).

**Figure 4:**
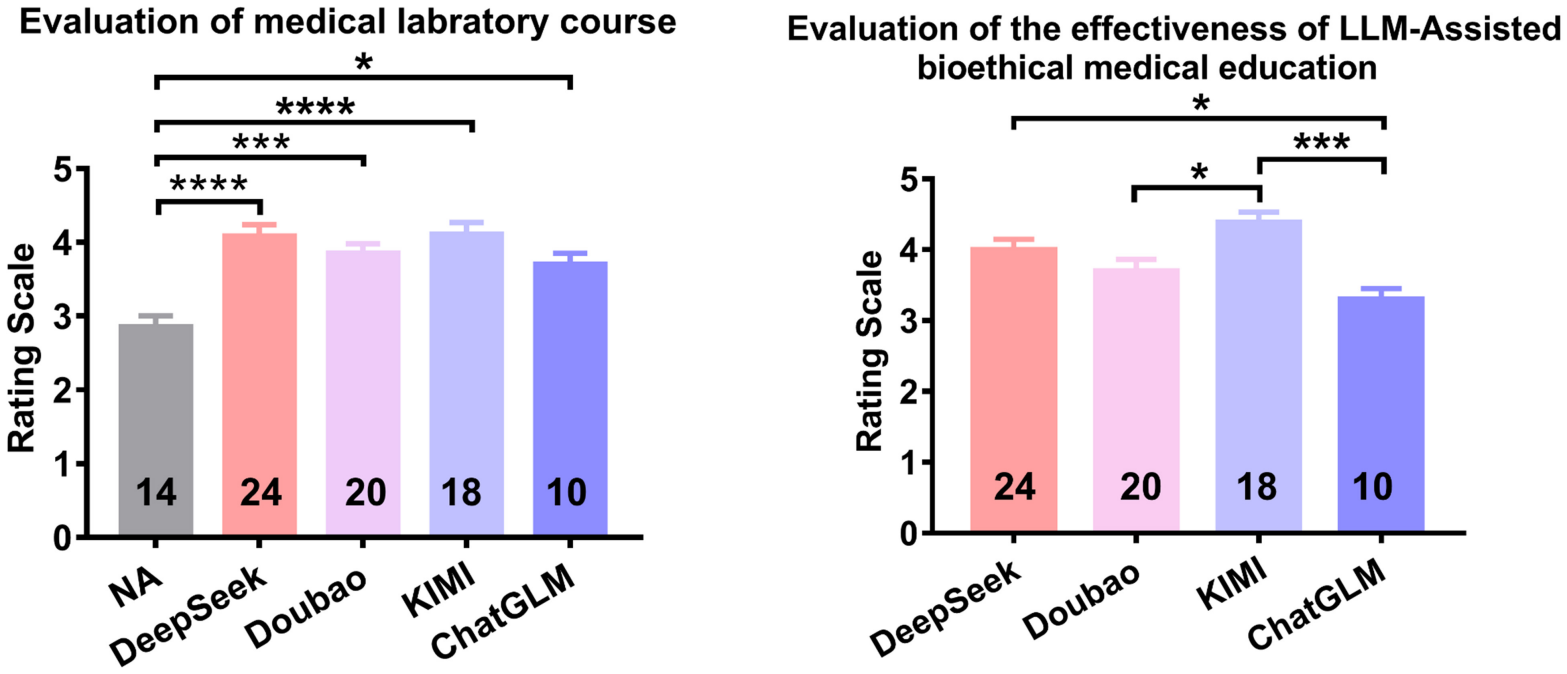
Pooled evaluation of medical laboratory course and effectiveness of LLM-assisted bioethical medical education among all participants (one-way ANOVA with *post-hoc* Tukey's test, *p < 0.05, **p < 0.01, ***p < 0.001, ****p < 0.0001).

In addition to students' self-assessments of their own report performance and instructor grading of laboratory reports, we conducted one-way ANOVA with Tukey's *post-hoc* tests to analyze two overall indicators: students' overall evaluation of the cell biology laboratory course and their overall assessment of the effectiveness of LLM-assisted bioethical medical education. The statistical results and relevant metrics are presented in below (Figure 4 and Tables 3–5).

**Table 3:** Summary of the statistical metrics.

**Summary of one-way ANOVA results and statistical metrics for the two research questions**								
**Questionnaire item**	**Comparison**	**No. of groups (** ***k*** **)**	**Sample size (N)**	***F***	***p*** **-value**	**df1 (** ***k*** **-1)**	**df2 (** ***N*** **–** ***k*** **)**	*η^2^*
Evaluation of the medical laboratory course	One-way ANOVA with *post-hoc* Tukey's test	5	86	9.69	< 0.001	4	81	0.32
Evaluation of the effectiveness of LLM-assisted bioethical medical education	One-way ANOVA with *post-hoc* Tukey's test	4	72	6.61	< 0.001	3	68	0.23

**Table 4:** Pooled evaluation of the medical laboratory course among students from three majors.

**Pooled evaluation of the medical laboratory course among students from three majors**			
**Comparison**	**Mean difference**	**95% CI**	**Adjusted** ***p***
NA vs. DeepSeek	−1.23	−1.83 to −0.62	< 0.001
NA vs. Doubao	−0.99	−1.62 to −0.36	< 0.001
NA vs. KIMI	−1.25	−1.90 to −0.61	< 0.001
NA vs. ChatGLM	−0.84	−1.59 to −0.09	0.019
DeepSeek vs. Doubao	0.23	−0.31 to 0.78	0.757
DeepSeek vs. KIMI	−0.03	−0.59 to 0.54	>0.999
DeepSeek vs. ChatGLM	0.38	−0.30 to 1.06	0.519
Doubao vs. KIMI	−0.26	−0.85 to 0.33	0.728
Doubao vs. ChatGLM	0.15	−0.55 to 0.85	0.975
KIMI vs. ChatGLM	0.41	−0.30 to 1.12	0.496

^*^Post hoc comparisons were performed using Tukey's multiple comparisons test.

**Table 5:** Pooled evaluation of the effectiveness of LLM-assisted bioethical medical education among students from three majors.

**Pooled evaluation of the effectiveness of LLM-assisted bioethical medical education among students from three majors**			
**Comparison**	**Mean difference**	**95% CI**	**Adjusted** ***p***
DeepSeek vs. Doubao	0.30	−0.23 to 0.83	0.457
DeepSeek vs. KIMI	−0.39	−0.94 to 0.16	0.255
DeepSeek vs. ChatGLM	0.70	0.04 to 1.37	0.035
Doubao vs. KIMI	−0.69	−1.26 to −0.12	0.012
Doubao vs. ChatGLM	0.40	−0.28 to 1.08	0.420
KIMI vs. ChatGLM	1.09	0.39 to 1.79	0.001

^*^Post hoc comparisons were performed using Tukey's multiple comparisons test.

To further determine whether students from different majors hold differing overall views regarding the effectiveness of LLMs in facilitating the integration of bioethical principles into the cell biology laboratory course, we analyzed and compared the evaluations from students in the three majors separately. The detailed assessment scores are presented in Table 6 and are consistent with the overall evaluation patterns shown in the Sankey diagram (Figure 3).

**Table 6:** Assessment of the effectiveness of the four LLMs by undergraduate medical students from three different majors in incorporating bioethics education into the cell biology laboratory course.

**Undergraduate students' assessment**	**Majors of undergraduate students**	**DeepSeek Mean (SD)**	**Doubao Mean (SD)**	**KIMI Mean (SD)**	**ChatGLM Mean (SD)**
Evaluation of the medical laboratory course	Medical imaging technology	3.9 (0.8)	3.9 (0.4)	4.1 (0.7)	3.7 (0.6)
	Health surveillance and quarantine	4.0 (0.8)	3.7 (0.8)	4.0 (0.7)	3.7 (0.6)
	Medical prevention	4.4 (0.7)	4.0 (0.6)	4.2 (0.8)	3.8 (0.5)
Apprasing LLMs-supported bioethical medical education outcomes	Medical imaging technology	4.3 (0.7)	3.9 (0.7)	4.3 (0.8)	3.3 (0.6)
	Health surveillance and quarantine	4.0 (0.8)	3.6 (0.8)	4.4 (0.5)	3.3 (0.6)
	Medical prevention	3.8 (0.7)	3.7 (0.8)	4.5 (0.5)	3.3 (0.5)

^*^Quantitative scale: 5 = excellent (or extremely); 4 = good (or very); 3 = fair (or moderately); 2 = limited (or slightly); 1 = poor (or not at all).

### Students' evaluation of their mastery of bioethical skills in medical learning process

In this study, the medical cell biology laboratory session focused on “examining the chromosome of the mouse sperms.” During Phase I, the instructor introduced the course objectives, background knowledge, bioethical principles, and the experimental protocol. In Phase II, students performed the experiment and have the option to choose whether to use LLMs. Those using LLMs could search protocols, clarify critical steps, review relevant bioethical principles, and address concerns related to animal handling, including mouse anesthesia and tissue dissection. All students who agree to participate in this study were required to complete data collection, a lab report, and a questionnaire evaluating the learning approach (LLMs-assisted vs. non-assisted). The questionnaire was designed around five dimensions of bioethics-centered laboratory education: (1) integrating humanistic values into experimental design, (2) applying ethical principles to guide strategy selection, (3) collecting samples responsibly, (4) minimizing potential harm, and (5) balancing scientific rigor with respect for life. Students self-rated the extent to which using (or not using) LLMs supported each dimension while studying instructional materials on bioethical policies, such as the “Replacement, Reduction, Refinement (3Rs)” animal-use principles, and related ethical guidelines. According to the questionnaire results, KIMI received the highest overall rating from students. With strong academic text comprehension and effective handling of both Chinese and English scholarly sources, KIMI offered well-structured and detailed information across all five core topics. Its ability to display reference sources alongside the generated content further facilitated students' use of relevant information. In comparison, DeepSeek, Doubao and ChatGLM provided more limited content, likely due to less extensive or less multilingual training data. Overall, all four LLM-assisted groups scored higher than the non-LLM group. A summary of the results and statistic metrics are presented in Tables 7, 8.

**Table 7:** Structure and internal consistency of the bioethical competency self-assessment.

**Structure and internal consistency of the bioethical competency self-assessment**						
**Dimensions**	**Subscale of bioethical competencies**	**No. of items**	**Representative self-assessment item in the questionnaire**	**Medical imaging technology (**α**)**	**Medical prevention (**α**)**	**Health surveillance and quarantine (**α**)**
1	Ethical awareness in experimental design	4	Please rate your ability to integrate ethical considerations into experimental design	0.828	0.796	0.787
2	Ethical reasoning in research planning	4	Please rate your ability to propose ethically appropriate experiental strategies	0.748	0.885	0.711
3	Ethical compliance in experimental practice	4	Please rate your ability to collect samples in accordance with animal welfare guidelines	0.856	0.824	0.735
4	Ethical risk management	4	Please rate your ability to optimize experimental procedures to minimize potential harm	0.796	0.836	0.781
5	Respect for life	4	Please rate your ability to ensure experimental integrity while considering the value of life	0.845	0.729	0.733

**Table 8:** Students' self-assessment of efficacy of LLMs in improving ethical competencies in experimental medicine.

**Students' self-evaluated bioethical competencies**	**Majors of undergraduate students**	**Without LLM mean (SD)**	**DeepSeek mean (SD)**	**Doubao mean (SD)**	**KIMI mean (SD)**	**ChatGLM mean (SD)**
Designing experiments with humane principles	Medical imaging technology	2.2 (0.8)	4.5 (0.8)	3.9 (0.7)	4.6 (0.5)	3.7 (0.6)
	Health surveillance and quarantine	2.0 (0)	4.1 (0.4)	4.0 (0.8)	4.2 (0.8)	3.3 (0.6)
	Medical prevention	2.3 (0.5)	4.5 (0.8)	4.3 (0.5)	4.5 (0.5)	3.5 (0.6)
Formulating feasible and ethical approaches	Medical imaging technology	2.0 (0.6)	4.3 (0.5)	3.6 (0.5)	4.4 (0.5)	4.0 (1.0)
	Health surveillance and quarantine	2.3 (0.5)	3.9 (0.4)	3.9 (0.4)	4.4 (0.9)	3.7 (0.6)
	Medical prevention	1.3 (0.5)	4.3 (0.7)	4.5 (0.5)	4.8 (0.4)	3.8 (0.5)
Acquiring samples with care and ethical consideration	Medical imaging technology	1.5 (0.5)	4.4 (0.5)	3.9 (0.7)	4.3 (0.5)	4.0 (0)
	Health surveillance and quarantine	1.8 (0.5)	4.1 (0.6)	4.0 (0.8)	4.2 (0.4)	3.7 (0.6)
	Medical prevention	1.8 (0.5)	4.5 (0.8)	4.2 (0.4)	4.7 (0.5)	4.0 (0.8)
Improving workflow to minimize adverse impact	Medical imaging technology	1.7 (0.5)	4.3 (0.5)	3.7 (0.8)	4.1 (0.4)	3.3 (0.6)
	Health surveillance and quarantine	1.5 (0.6)	4.0 (0.8)	3.7 (1.0)	4.4 (0.5)	3.3 (0.6)
	Medical prevention	1.5 (0.6)	4.6 (0.5)	4.3 (0.5)	4.2 (0.4)	4.0 (0.8)
Safeguarding experimental integrity while honoring life	Medical imaging technology	2.2 (0.4)	4.4 (0.7)	4.0 (0.6)	4.4 (0.8)	3.7 (0.6)
	Health surveillance and quarantine	2.3 (0.5)	4.0 (0.5)	3.9 (0.4)	4.2 (0.4)	4.0 (1.0)
	Medical prevention	2.3 (0.5)	4.4 (0.5)	4.2 (0.4)	4.8 (0.4)	3.8 (0.5)

^*^Quantitative scale: 5 = excellent; 4 = good; 3 = fair; 2 = limited; 1 = poor.

### Participants' perceived attitudes and concerns about LLMs in bioethics-oriented medical education

Students were then asked to evaluate the content quality and overall effectiveness of the LLMs-assisted bioethics-centered approach in the biomedical laboratory course. Results showed that students generally favored this instructional method and provided positive feedback on the LLM-assisted sessions. At the same time, students expressed concerns about the use of LLMs in bioethics-related medical education, centering on three issues: uncertainty when addressing complex biomedical questions, bias in interpreting bioethical principles, and the potential reduction of independent thinking. Specifically, students reported that Doubao often generated responses without proper citation, and ChatGLM relied heavily on non-peer-reviewed sources such as Baidu (a widely-used Chinese search engine) and ZhiHu (a well-known Chinese forum), which heightened concerns about accuracy and objectivity. In contrast, KIMI distinguished itself by providing responses supported by academic journals, scholarly forums, and knowledge platforms, which increased students' confidence in the platform's reliability and academic value in integrating bioethical principles into undergraduate medical training (Table 9).

**Table 9:** Students' perspective on LLMs in ethics-oriented undergraduate medical education.

**Insights into LLMs in ethics-oriented medical education**	**Majors of undergraduate students**	**DeepSeek mean (SD)**	**Doubao mean (SD)**	**KIMI mean (SD)**	**ChatGLM mean (SD)**
Academic uncertainty	Medical imaging technology	3.5 (0.5)	3.7 (0.5)	3.6 (0.5)	3.7 (0.6)
	Health surveillance and quarantine	3.4 (0.5)	3.9 (0.4)	3.4 (0.5)	3.7 (0.6)
	Medical prevention	3.6 (0.5)	3.8 (0.4)	3.7 (0.5)	4.0 (0)
Prejudices in academia	Medical imaging technology	2.8 (0.5)	2.9 (0.7)	2.4 (0.5)	2.7 (0.6)
	Health surveillance and quarantine	2.8 (0.7)	2.7 (0.5)	2.4 (0.5)	2.7 (0.6)
	Medical prevention	2.6 (0.7)	2.8 (0.4)	2.5 (0.5)	2.8 (0.5)
Promotion rather than impairment of independent thinking	Medical imaging technology	3.8 (0.5)	3.7 (0.8)	3.9 (0.7)	3.7 (0.6)
	Health surveillance and quarantine	3.6 (0.5)	3.7 (0.5)	3.6 (0.5)	3.7 (0.6)
	Medical prevention	3.5 (0.5)	3.7 (0.5)	3.5 (0.5)	3.8 (0.5)

^*^Quantitative scale: 5 = excellent (or extremely); 4 = good (or very); 3 = fair (or moderately); 2 = limited (or slightly); 1 = poor (or not at all).

### Comparing LLM performance on students' representative questions about bioethical principles in medical education

To compare the generated content and the feature of each LLMs in the context of medical cell biology laboratory course, we selected several representative questions from students and asked each LLM to provide informed responses. The first question addressed bioethical policies, the second examined the balance between scientific objectives and respect for life, the third explored professional values in a medical career, the fourth investigated emotional reflection in clinical challenges, and the fifth focused on educational innovation. All four LLMs provided basic coverage of these topics. Notably, KIMI, trained on a multilingual corpus with diverse literature and scientific resources, consistently produced detailed, structure, and contextually solid responses, often accompanied by references. These features enhance transparency, traceability, and academic rigor, highlighting KIMI's potential to support advanced bioethics-centered medical education. Detailed responses from each LLM are presented below (Table 10).

**Table 10:** Typical questions posted by students in the medical laboratory course and corresponding responses from each LLM.

**How have you made use of LLMs to tackle specific problems or challenges encountered in basic medical experiments?**				
**Common questions that students ask LLMs**	**DeepSeek**	**Doubao**	**KIMI**	**ChatGLM**
1. Policies: How is the typical animal ethics approval process conducted in universities or academic institutes?	The process involves researchers submitting a detailed proposal to an Institutional Animal Care and Use Committee (IACUC). The IACUC reviews the protocol to ensure ethical justification, compliance with regulations, and minimization of animal suffering.	First, submit a detailed research plan. Then, the ethics committee reviews it. Approval is given if it meets animal welfare and ethical standards.	In universities, researchers submit detailed proposals to an Institutional Animal Care and Use Committee (IACUC). The committee reviews these proposals for ethical compliance, ensuring minimal animal distress and scientific necessity. Amendments may be requested. Once approved, researchers must adhere strictly to the approved protocols.	Animal ethics approval involves submitting a detailed research proposal to an institutional committee. It reviews the study's necessity, animal welfare standards, and compliance with legal and ethical guidelines before granting or denying approval.
2. Scientific purpose vs. Life respect: Which pain control methods are advised for testicular sampling in mice?	For testicular sampling in mice, multimodal analgesia is advised. This typically includes a systemic analgesic like meloxicam (NSAID) and/or buprenorphine (opioid), often combined with local anesthetics such as lidocaine or bupivacaine at the incision site.	Before testicular sampling, use isoflurane or ketamine-xylazine for general anesthesia. Afterward, administer long-acting analgesics like buprenorphine and provide a warm, stress-free recovery environment.	For testicular sampling in mice, recommended pain control methods include the use of local anesthetics such as bupivacaine and systemic analgesics like meloxicam. These methods can effectively reduce pain and distress in mice undergoing such procedures.	Recommended pain control methods for testicular sampling in mice include local anesthetics (e.g., bupivacaine), systemic analgesics (e.g., meloxicam or buprenorphine), and general anesthesia (e.g., isoflurane). These methods minimize discomfort and ensure animal welfare during and after the procedure.
3. Professional values: What factors should be taken into account during animal euthanasia? How might exposure to animal experiments influence my professional values?	Ethical euthanasia requires justifying the decision, ensuring unconsciousness precedes death, and minimizing distress. Exposure to animal research can profoundly shape one's professional values regarding ethical responsibility, the sanctity of life, and scientific rigor.	For euthanasia, consider methods' humaneness, animal species, and legal requirements. Exposure may reshape views on life, ethics, and medical responsibility.	During animal euthanasia, consider factors such as the method's humaneness, speed, reliability, and the animal's species and age to minimize suffering. Exposure to animal experiments may influence professional values by reinforcing the importance of ethical conduct, respect for animal welfare, and the need for scientific necessity and justification.	Key factors in euthanasia include method appropriateness, animal welfare, legal and ethical guidelines, and personnel training. Exposure to animal experiments can deepen commitment to ethical research, humane treatment, and scientific integrity, shaping professional values centered on responsibility and compassion.
4. Emotional reflection: How could participation in animal experiments affect my mental health, and what support resources are available if I experience distress?	Participation can induce moral distress, anxiety, or guilt. Support resources are crucial and typically include access to institutional counseling services, peer support groups, and ethics committees to help researchers process these complex emotional experiences.	Performing animal experiments may cause guilt and stress. Seek support from advisors, university counseling services, or fellow students.	Participation in animal experiments may cause mental distress due to ethical conflicts and exposure to animal suffering. If you experience distress, consider seeking support from colleagues, professional counseling services, or support groups specifically for researchers dealing with similar issues.	Participation in animal experiments may induce stress, guilt, or moral distress. Support resources include counseling services, peer support groups, and mental health programs. Institutional animal care committees often provide guidance to promote psychological wellbeing and ethical resilience.
5. Educational innovation: Can LLMs-supported simulations contribute to minimizing animal use in our laboratory courses?	Yes, LLM-supported simulations can significantly reduce animal use. They provide sophisticated, repeatable models for teaching physiological and pharmacological principles, thereby aligning educational objectives with the ethical framework of the 3Rs (Replacement, Reduction, Refinement).	LLM-based simulations can mimic real-world scenarios, enabling students to practice, thus potentially reducing animal use in lab courses.	LLM-supported simulations can effectively minimize animal use in laboratory courses by offering realistic, interactive, and repeatable learning experiences. They allow students to practice techniques and understand complex concepts without compromising on educational quality, thus aligning with the 3Rs principles (Replacement, Reduction, Refinement).	LLM-supported simulations can significantly reduce animal use in laboratory courses by providing realistic, interactive alternatives for teaching experimental techniques and physiological concepts. This approach adheres to the 3Rs principle, enhancing ethical standards while maintaining educational efficacy.

## Discussion

Compared with existing studies, our results indicate that among the most robust and widely used LLMs in China, KIMI and DeepSeek demonstrate favorable performance in integrating bioethical principles into the cell biology curriculum. These findings highlight the potential of these platforms not only to enhance bioethics-centered learning in this context but also to inform broader applications and adoption across undergraduate medical education (see Figure 4 and Table 6).

On one hand, LLMs rely on the breadth and diversity of their training data, and their ability to generate accurate and contextually relevant outputs depdns on the quality of these datasets. Current studies have mainly focused on one of the most powerful LLMs, ChatGPT; however, educational studies on LLMs trained on Chinese-language databases remain limited, and their suitability for routine use in China is not well-established (29). On the other hand, due to the restricted accessibility of ChatGPT in China, there is an urgent need to develop and provide LLM alternatives that are better suited for localized use in China (30). Specifically, identifying the most effective LLMs model for undergraduate medical training in China could prominently enhance learning quality in multiple realms.

Although prior studies have mainly focused on LLMs-assisted enhancement of medical knowledge, relatively little attention has been given to integrating medical humanities, bioethical principles, and life care into foundational curricula (31, 32). Early exposure to medical ethics benefits first-year students by providing conceptual grounding and emotional preparedness for future challenges (33). Given their reliance on instructor guidance, we implemented an approach combining traditional teaching with LLM-assisted bioethics learning, allowing students to deepen understanding and explore case-specific solutions during experiments, thereby enhancing personalized learning and outcomes. Systematic investigation of China-developed LLMs in integrating bioethical awareness remains scarce. This study provides empirical evidence and practical insights on the potential of Chinese LLM platforms to embed bioethical principles into foundational medical education. Trained on extensive Chinese and English datasets related to bioethics, the four LLMs evaluated in this study demonstrated strong performance in providing effective references that helped undergraduate students develop bioethical sensitivity during medical training.

Beyond the integration of bioethics into the curriculum, students face ethically and emotionally demanding experiences in laboratory and clinical settings. Earlier research on bioethical education has mainly focused on the ethical treatment of experimental subjects. However, students themselves may experience moral distress, ethical stress, and psychological burden in ethical decision-making, manifesting as anxiety, guilt, fear, or internal conflict (34). In this study, many students exhibited such responses when handling experimental animals for the first time, particularly when required to sacrifice them for sample collection. These reactions mirror challenges they may later face in high-stakes clinical settings, such as critical care or end-of-life situations. We therefore explored whether LLMs could guide students through these ethically and psychologically challenging scenarios, alleviate moral and ethical distress, and foster resilience. Trained on extensive Chinese and English datasets related to bioethics, the four LLMs evaluated in this study demonstrated strong performance in providing effective references that helped students develop bioethical sensitivity, offering valuable empirical and practical support for integrating ethics into foundational medical education.

Previous studies have found it challenging to address the potential drawbacks of applying LLMs in the context of medical education in China (35, 36). To fill this gap, we collected students' opinions on the limitations they encountered while using LLMs in laboratory coursework and proposed several strategies to mitigate these issues. The main concerns reported were related to academic uncertainty, academic bias, and impairment of independent thinking. To address these concerns, we suggest the following approaches. First, students should verify the information provided by LLMs by cross-checking it with original academic sources to ensure accuracy and reliability. For example, there is broad international consensus that germline editing of human embryos remains ethically unacceptable under current conditions (37). By contrast, somatic gene-editing therapies targeting adult patients, such as base-editing therapies for transfusion-dependent β-thalassemia in China (38) and CRISPR-based therapies approved by the U.S. Food and Drug Administration (39), have different ethical, regulatory, and clinical considerations depending on the country. Students must therefore interpret LLM-generated information in the context of local policies and regulations rather than adopt it uncritically. Second, students should be encouraged to participated in discussions and exchange viewpoints on specific scientific questions, thereby fostering their independent thinking skills. Prior research has demonstrated that proactively engaging in research activities enhances both students' interest in the subject matter and their critical thinking skills (40, 41). Therefore, through this approach, LLMs can stimulate creative thinking rather than hinder it, helping students learn how to apply bioethical principles in the medical field, and providing conceptual and psychological preparation for the students' future role in developing these principles.

Building on these findings, and as one of the most distinctive contribution of this study, we implemented a closed-loop, teacher-student-LLM research model integrating instruction, active student engagement, and bilateral evaluation. This approach not only provides a comprehensive understanding of the students' learning process but also allows bioethics-centered education. Building on the class-based data and student feedback obtained through this model, teachers are thus better positioned to further refine both the methodology and practical implementation of the educational reform project. Specifically, LLMs-assisted instruction can be designed to help students navigate ethical issues that arise during their study of basic medical courses, offering timely, accurate, inclusive, and personalized support. This strategy not only strengthens the overall effectiveness of the closed-loop educational system but also aligns with recent perspectives in the field of LLMs-assisted education, which highlight the importance of integrating continuous feedback mechanisms and promoting equitable and high-quality learning experiences (42).

## Strength and limitations

This study has several notable strengths. First, it rigorously investigates how mainstream LLMs can support bioethics-oriented education in undergraduate medical curricula. Second, it involved students from three representative medical majors—Medical Imaging Technology, Medical Prevention, and Health Surveillance and Quarantine—broadly reflecting the wider medical student population. Third, given medicine's specialization and rapid evolution, the study provides a foundation for applying LLMs in ethics-focused biomedical education and clinical training, potentially fostering well-rounded professionals with advanced competencies.

However, several limitations of the study should be acknowledged. The selected majors had relatively small class sizes, which restricted the number of participating students. Only four LLM platforms—DeepSeek, Doubao, KIMI, and ChatGLM—were evaluated, reflecting limitations in availability and cohort size. In addition, the rapid iteration of LLMs means that the findings should be considered preliminary, providing initial insights into incorporating life-centered perspectives in Chinese undergraduate medical education. On one side, this study is limited by its quasi-experimental design with student self-selection. Individual characteristics, such as motivation, prior experience with digital tools, or attitudes toward LLM-assisted learning, may have influenced outcomes. Baseline data indicate that participants across the three majors were broadly comparable in academic background, course exposure, demographics, and pre-course interest and experience with LLMs, which may partially mitigate this concern. Nevertheless, unmeasured confounding cannot be fully excluded, and future randomized or matched designs would strengthen causal inference. On the other side, selection bias and limited generalizability remain concerns, as all participants were drawn from a single institution and a single cell biology laboratory course. In addition, all students shared the same learning environment, making informal interactions across groups possible, which could have influenced engagement. Future studies could explore multi-institutional samples or separate course sections to address these issues. Furthermore, performance bias may have occurred because students were aware of their group assignments and access to LLMs, potentially affecting motivation or expectations. Moreover, measurement bias is possible, as learning outcomes and ethical understanding were assessed through self-reported questionnaires, which are susceptible to social desirability and response expectancy effects. Incorporating objective or performance-based assessments would enhance robustness in future research. In addition, while some differences were observed among groups using different LLMs, the study was not designed for definitive comparisons. These observations should be interpreted cautiously as preliminary insights into how structured LLM use may support engagement with bioethical topics in laboratory-based medical education.

These findings suggest that LLM-assisted approaches have the potential to support bioethical learning in undergraduate medical courses, while remaining exploratory and context-specific. Despite limitations in sample size, platform scope, and the rapid evolution of LLMs, the results offer preliminary guidance for integrating life-centered perspectives into medical education. Further studies with larger cohorts and broader disciplinary coverage are needed to validate and extend these insights.

## Conclusion

LLM-assisted technologies may offer a supportive role in undergraduate medical education by complementing traditional instruction and engaging students with complex ethical issues. This study explored the use of four widely used LLM platforms in China (DeepSeek, Doubao, KIMI, and ChatGLM) within a basic medical laboratory course, focusing on their potential contribution to bioethical understanding, ethical skill development, and students' perceptions of LLMs in medical learning contexts. The findings suggest that LLM-assisted learning can be integrated into laboratory-based instruction and may help facilitate students' engagement with bioethical topics. While some platforms, such as KIMI and DeepSeek, were associated with relatively more positive outcomes in this specific educational setting, these observations should be interpreted cautiously within the exploratory scope of the study. The results are not intended to indicate the superiority of any particular tool, but rather to provide preliminary insights into how structured use of LLMs might support ethical reflection and learning processes in medical education. Overall, this exploratory educational study provides initial evidence that LLM-assisted approaches can enrich bioethics-related learning experiences in basic medical training. The findings are context-specific and have limited generalizability beyond the institution and the four Chinese LLM platforms examined. Further research using larger samples, more rigorous designs, and diverse educational contexts is needed to better understand the potential benefits and long-term value of LLM-supported instruction. Such efforts may inform the responsible and pedagogically sound integration of LLM technologies into future medical curricula.

## Data Availability

The datasets generated or analyzed in this study are available from the corresponding author on reasonable request.
